# Let there be light: Artificial light cues improve early life ramp use of laying hen chicks in a commercial aviary

**DOI:** 10.1016/j.psj.2025.105546

**Published:** 2025-07-07

**Authors:** Alex Johny, Dominik Guggisberg, Michael Jeffery Toscano, Ariane Stratmann

**Affiliations:** a Center for Proper Housing: Poultry and Rabbits (ZTHZ), Division of Animal Welfare, VPH-Institute, University of Bern, Burgerweg 22 3052, Zollikofen, Switzerland; b Graduate school of Cellular and Biomedical Sciences, University of Bern, Bern 3012 Switzerland; c Agroscope, Food Microbial Systems 3003 Bern, Switzerland

**Keywords:** Behavior, Rearing phase, Bone biomechanics, Predispositions

## Abstract

In rearing aviaries, the provision of ramps has been found to facilitate the early utilization of elevated structures and increase inter-tier transitions. To maximize the welfare benefits associated with ramp use during early life, we investigated whether a moving light cue could increase early-life ramp use in laying hen chicks and subsequently improve bone biomechanical properties at a later age. The light cue was initially developed and tested in experimental settings, and this study aimed to validate the results from the experimental setting in a commercial setting. A total of 4800 Dekalb white birds were housed in eight pens (600 birds/pen) from one until 17 weeks of age (WoA) in a semi-commercial rearing barn. Ramps were provided in all pens, with half of the pens equipped with LED strips that provided intermittent light cues throughout the day (LED group), while the other half served as control pens without any light cues (CON). The number of inter-tier transitions and active use (e.g., walk/run, wing-assisted incline running) of ramps that did not result in a transition were recorded by scan sampling videos at multiple time points, when the cues were on as well as when they were off in LED pens. Same observation times were used in CON as well, although they did not have light cues. Additionally, bone biomechanical properties were assessed in tibiae and humeri collected from a subset of 160 birds (20 birds per pen) at 15 WoA. The analysis revealed that birds from the LED group exhibited more transitions and active use of ramps when the light cues were on compared to when they were off, as well as compared to the CON group (both cue-on and cue-off periods) throughout the observation period (until 10 WoA). No differences in bone biomechanical properties were observed between the CON and LED groups. These findings demonstrate that artificial light cues are suitable as a commercially viable tool to encourage vertical locomotion in laying hen chicks.

## Introduction

Over the past few decades, commercial laying hen housing has been transitioning from cage systems to cage-free systems in many parts of the world (Bessei, 2018). In the European Union, multi-tier aviaries are favored for both rearing and production phases, as they allow for better utilization of available barn space (Janczak and Riber, 2015). Aviaries are considered better for the welfare of the birds compared to cages (Lay et al., 2011), but they still present welfare challenges due to their height and complex structure that are difficult to navigate (Scott and Parker, 1994; Scott et al., 1997). The height and complexity of aviaries are also linked to falls and collisions (Campbell et al., 2016; Stratmann et al., 2019), which are believed to contribute to the incidence of keel bone fractures (Toscano et al., 2020), a major welfare concern in the laying hen industry (Harlander-Matauschek et al., 2015).

Use of ramps, a structure that provides a continuous path between different aviary tiers, has been shown to increase movement between tiers and decrease keel bone damage in the laying phase (Stratmann et al., 2015; Toscano et al., 2024). Additionally, early exposure to ramps during rearing encouraged the use of elevated surfaces (Stratmann et al., 2022), improved movement between aviary tiers (Norman et al., 2021; Stratmann et al., 2022), reduced hesitancy in inter-tier transitions (Norman et al., 2018), and decreased keel bone fractures at 40 weeks of age (Norman et al., 2021). Norman et al. (2021) hypothesized that the decrease in keel fractures could be attributed to improved skeletal properties resulting from early access to vertical surfaces facilitated by the ramps. Previous studies have demonstrated that load-bearing exercises, such as those facilitated by multi-tier aviaries during rearing, positively influence skeletal properties (Casey-Trott et al., 2017a, 2017b; Pufall et al., 2021; Regmi et al., 2015; Rentsch et al., 2024).

Given the welfare benefits associated with early-life ramp use, we previously conducted two small-scale experiments to investigate the use of artificial cues to encourage early life ramp use in laying hen chicks (Johny et al., 2023). We tested multiple cues that leveraged the innate predispositions of domestic chicks, that aid in social interactions (Bolhuis, 1991; Miura and Matsushima, 2016; Rosa-Salva et al., 2019), foraging behavior (Hogan, 1973) and predatory instincts (Fernández-Juricic, 2004). The results of our experiments showed that the light cues simulating the motion of a small moving object were particularly effective in improving ramp use. The light cues were designed to utilise several predispositions of laying hen chicks, namely the inherent preference to red and blue colours (Ham and Osorio, 2007), signs of animacy such as self-propulsion (Mascalzonia et al., 2010) and speed changes (Rosa-Salva et al., 2016), as well as foraging related behaviours such as pecking small particles (Hogan, 1973). However, it is important to test the cues in commercially relevant settings for external validity and applicability for the industry.

Commercial laying hen houses differ substantially in animal numbers, have complex environments and larger flock sizes compared to most experimental settings. The differences in flock size have been shown to affect various behaviors such as aggression (Estevez et al., 2003, 2002), social relationships (D’Eath and Keeling, 2003), perch use (Newberry et al., 2001) and space utilization (Liste et al., 2015). Commercial farms also differ in animal management practices and environmental parameters, such as ammonia concentration and litter quality. In consideration of these concerns, it is important to evaluate results of small-scale testing in commercially relevant settings (Dawkins, 2012).

Our current experiment aimed to evaluate the results obtained from the controlled experimental setting within a commercial barn. We investigated whether a light cue that simulated the movement of a small object would increase the use of ramps in the early life of laying hens in a semi-commercial rearing barn. Additionally, we assessed whether the increased ramp use induced by the moving light cue influenced biomechanical properties of the tibia and humerus. We hypothesised that the use of light cues would lead to a greater number of inter-tier transitions and more active use of ramps and consequently, improve bone properties in pullets.

## Materials and methods

### Ethics

The experiment received approval from the Veterinary Office of the Canton of Bern, Switzerland (BE 106/19) and was conducted in accordance with all federal and cantonal regulations ensuring the ethical treatment of animals involved in research.

### Animals and housing

**Fig. 1:**
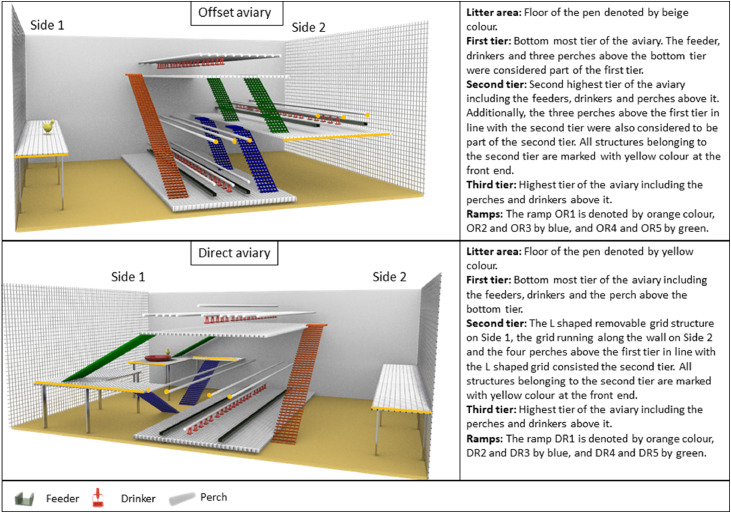
The schematic representation of the two aviary systems, description of aviaries tiers and ramp installation.

For this experiment, 4,800 Dekalb White birds were reared from one day of age (DoA) until 17 Weeks of Age (WoA) in a semi-commercial rearing barn at the Aviforum in Zollikofen, Switzerland. The barn was divided by a central wall, with each side containing two different multi-tiered rearing aviary systems. Each side was further partitioned into four pens, resulting in a total of eight pens (four pens per barn side). The pens were populated with one day old chicks, with each pen housing 600 birds. The four pens on one side of the barn were fitted with the Inauen Natura AZ aviary system (Inauen AG, Appenzell, Switzerland, Fig. 1) which provided a total usable space (i.e., tier and litter area) of 42.74 m^2^ (pens 1-3) and 41.61 m^2^ (pen 4). The dimensions of each pen were approximately 4.80 × 3.50 × 2.35 m (*L*× *W*× *H*). The aviary consisted of two tiers vertically stacked (referred to as the Direct aviary from henceforth), with feeding chains and nipple drinkers on each tier. Perches (diameter = 0.34 m) were provided 0.5 m above both tiers and ran through the length of the pens. Raised platforms were installed in the litter area on each side of the Direct aviary, and a feeder plate was placed on one of the raised platforms. A pop hole connected each pen to a winter garden (covered outdoor area) measuring 4.90 *m*× 2.55 *m*× 2.35 m (*L*× *W*× *H*). The winter garden for each pen was separated by wire mesh, which prevented the mixing of birds from different pens. Pens on the other side of the barn were fitted with the Landmeco Harmony aviary (Globogal AG, Lenzburg, Switzerland, Fig. 1). Each pen provided a total usable space of 40.99 m^2^ (*L*× *W*× *H*: 4.90 × 4.55 × 2.20 m). The aviary featured three vertically stacked tiers arranged in an offset fashion (referred to as Offset aviary henceforth). The first and second tiers had feeding chains, while drinkers were available on all tiers. Perches (diameter = 0.34 m) ran through the length of the aviary and were provided on all tiers. Wood shavings were spread on the floor as litter. Raised platforms were also incorporated into each pen along one of the sidewalls. Similar to the Direct aviary, a pop hole connected each pen to a winter garden (L X *W*× *H*: 4.95 × 3.45 × 2.20 m). The winter garden in all pens (both Direct and Offset aviaries) included an A-frame structure with five wooden perches and wood shavings on the floor. From six WoA until the end of the rearing phase at 17 WoA, all birds had access to the winter garden between 10:00 and 16:00. The barn used in our experiment differed from a true commercial barn, in terms of the division of the barn into pens as well as the addition of elevated metal grids. Also, the barn had two different aviaries which is not a common commercial practice.

Artificial light was provided according to standard rearing management practices for Dekalb White birds (Supplement 1). The birds received 24 hours of light for the first two days, which was gradually reduced to nine hours by the 10th WoA and maintained until the 16th WoA. In the 17th WoA the birds received 10 hours of light. The lighting program included a one-minute transition phase at dawn and a five-minute dimming phase at dusk. Daylight was also accessible through automatically controlled windows. The birds were fed with starter feed from one to nine WoA, followed by pullet feed from nine to 17 WoA. The birds were populated at one DoA and confined to the first tier of both aviaries until six DoA. They were provided access to the entire aviary through custom made ramps (described below) that connected the various elevated structures and tiers within the aviaries from six DoA.

### Experimental design

All pens were fitted with custom-made ramps of a metal grid (0.65 m × 0.25 m (*L*× *W*)) in a way that the configuration and number of ramps were comparable between both aviaries, even though they differed in their structure. Thickness of the metal grids were 3 mm for all ramps. In the Offset aviary, there was a single ramp (Offset Ramp 1 (OR1): 2.26 × .25 m, Fig. 1) connecting the first and third tiers. Additionally, a pair of ramps (OR2 and OR3: 1.14 × .25 m, Fig. 1) connected the first and second tiers. Another pair of ramps (OR4 and OR5: 1.20 × .25 m, Fig. 1) connected the second and third tiers in the Offset aviary. Additional small ramps (0.59 × 1.22 m (*L*× *W*)) ran from the litter area to the first tier from six until 22 DoA to help chicks move between the first tier and the litter area. In the Direct aviary, there was a single ramp (Direct Ramp 1 (DR1): 2.26 × .25 m, Fig. 1) connecting the litter area to the second tier. Additionally, a pair of ramps (DR2 and DR3: 0.92 × .25 m, Fig. 1) connected the litter area to the raised platforms, and another pair of ramps (DR4 and DR5: 1.24 × .25 m, Fig. 1) connected the raised platforms to the second tier. The movement of the chicks from the litter area to the first tier was further facilitated by the presence of wooden bars that served as steps between the litter areas from six until 22 DoA.

Two pens per aviary system were selected as the light treatment groups (LED) and the other two served as control pens (CON). The ramps in both aviaries were placed in a manner to obtain comparable use of ramps between the aviaries allowing combining the treatment pens across the aviaries for analysis (*n*= 4). The light cues were provided using LED strips (Seeed Studio, WS2813) fitted vertically along the centre of the ramps (Video supplement 1). The length of the LED strip was adjusted according to the length of the ramps (Offset: OR1 – 1.70 m, OR2, OR3, OR4 and OR5 – 1.00 m, Direct: DR1 – 1.70 m, DR2, DR3 – 0.80 m, DR4, DR5 – 1.00 m). Each LED strip had 60 LED bulbs/m. Two adjacent bulbs were programmed to go on and off sequentially to simulate the movement of a small object of red colour ((RGB = [225, 48, 0]) moving up and down the ramp. The speed of flashing LED bulbs changed every minute following a sequence of 5.5, 12.5, 25.0, and 8.3 cm/s. The cues were applied in bouts of eight minutes with the sequence repeating twice within a bout. The cue bouts were dispersed throughout the day and accounted for 8.33 % of the day length at one WoA, which was gradually reduced to 5.93 % by 10 WoA. The number of cue bouts was reduced to account for the reduction in day length period stipulated by rearing management practices. The cues were not applied for 60 minutes after lights on and 135 minutes before lights off as maximum feed intake happens within these periods (Savory, 1980). The cues were also not applied from 08:00 to 11:00 when the routine management took place. The CON pens did not have LED strips attached to the ramps, meaning the control and treatment pens looked different even when the cues were not applied.

The pens were separated by wire mesh and thick plastic sheets were attached to obstruct the view of the ramps in neighbouring pens. Pens were not fully visually isolated to ensure air circulation within barn sides and within each pen as heaters and ventilators were positioned at the rear end of each barn side. The birds from each pen could thus see and hear the birds from other pens, but the direct view of the ramps and hence the cues were obstructed. We also assigned the treatment pens in blocks to minimize the cross-treatment influences between pens (Supplement 2).

### Data collection

***Ramp use Behavior.*** Each pen was equipped with two cameras (Samsung SCO-2080R, IR, Samsung Techwin CO., Korea) positioned on both sides of each pen and connected to a customizable recording software platform (Multieye Hybrid Recorder Version 2.3.1.8, Artec Technologies AG, Diepholz, Germany) to record the birds’ behavior on the ramps.

We quantified the behavior of the birds at various DoA (8, 11, 16, 23, 30, 37, 44, and 56 DoA) from recorded videos, as specified in the ethogram in Table 1. To account for the influence of changing day length on birds' activity patterns, six observation bouts (three bouts when light cues were on and three when cues were off) were selected each day for analysis. The whole light period was divided into three equal parts where only the first light cue bout that occurred in each period was assessed. We analysed the behavior of the birds during the second to sixth minute (a total of 5 minutes) of the selected light cue bout, resulting in a total of 15 minutes of analysis for the cue-on period per pen side and DoA. We also analysed the behavior of the birds when the cues were not operating in pens of each treatment from the 42nd to the 46th minute that followed the analysed light cue bout. For instance, if the light cue bout at 07:00 was chosen for analysis, we additionally analysed the behavior of the birds from 07:42 to 07:46 when the cues were not applied. CON pens were observed during the same time slots as LED pens (both cue-on and cue-off) to ensure temporal consistency of observation periods across treatments. The terms cue-on and cue-off when used for the CON group refers to the observations time periods corresponding to cue-on and cue-off periods in the LED group, respectively. Technically, there should be no difference between cue-on and cue-off periods within the CON group, as no light cues were applied. However, results from our earlier small-scale experiments (Johny et al., 2023) showed increased ramp use in control pens during periods when cues were active in adjacent treatment pens despite all pens being visually isolated. Hence for this experiment, we decided on including the cue-off period observations for CON as well as for the analysis as we thought it might be the closest to the true baseline ramp use in our study. Ideally, cue-off periods in LED and cue-on and cue-off periods in CON are the same and serve as baseline ramp use. However, the increase in ramp use in cue-on periods in the LED pens could increase ramp use in CON pens if there is social facilitation. Although direct visual access to ramps between pens were prevented, chicks could see parts of the neighbouring pens. Also, birds could hear each other, which can also influence ramp use behaviour of the CON birds when cues are on in the LED pens. The cue-off periods in LED pens might also not be true baseline, if the light cues lead to a shift in the baseline ramp use rather than an absolute increase in ramp use. For instance, an increase in ramp use during cue-on periods could be followed by a compensatory decrease during cue-off periods. In that case, the highest ramp use would be during the LED cue-on period, followed by cue-on and cue-off periods in CON pens, and the lowest for cue-off periods in LED pens. Also, the total ramp use (cue-on + cue-off) in both treatments would be the same. Thus, comparing cue-on and cue-off conditions across both treatments allows us to disentangle the factors contributing to increased ramp use and determine if light cues are responsible for this increase.

**Table 1:** Ethogram of the behaviors analysed from videos.

Behavior		Ramp^1^	Transition category
Transitions: Bird moves from one tier to another^2^	Using ramps: Bird moves from one tier to another by walking, running or WAIR^3^ on ramps	OR1	Transition from first tier/litter to third tier or vice versa
			Transition from first tier/litter to second tier or vice versa
			Transition from second to third tier or vice versa
		OR2 and OR3	Transition from first tier/litter to second tier or vice versa
		OR4 and OR5	Transition from second to third tier or vice versa
		DR1	Transition from first tier/litter to third tier or vice versa
			Transition from first tier/litter to second tier or vice versa
			Transition from second to third tier or vice versa
		DR2 and DR3	Transition from first tier/litter to second tier or vice versa
		DR4 and DR5	Transition from second to third tier or vice versa
	Without using ramps: The bird jumps or flies from one tier to another without using ramps	Offset Side 1	Jump/fly from third to first tier/ litter
		Offset Side 2	Jump/fly from second to third tier or vice versa
		Direct Side 1	Jump/fly from second to third tier or vice versa
		Direct Side 2	Jump/fly from third to first tier/ litter
Active behaviors on ramps without transition^4^: The bird runs, walks, or performs WAIR on the ramp which does not result in a transition.		OR1	
		OR2 and OR3	
		OR4 and OR4	
		DR1	
		DR2 and DR3	
		DR4 and DR5	

1 Refer to Fig. 1 for ramp definitions.

2 Direction of transition was not recorded.

3 WAIR – wing-assisted incline running.

4 All active behaviors that a bird performed on a ramp from entering the ramp to exiting the ramp were counted as one event. For example, if the bird entered the ramp by running onto it, stayed on the ramp for ten seconds and exited the ramp by WAIR without a tier change, it was counted as one active behavior.

***Bone biomechanics.*** At 15 WoA, 20 birds per pen were chosen arbitrarily from different pen areas (i.e., eight from the litter area, four each from the first, second and third tiers) for bone biomechanical analysis (total = 20 birds/pen, 80 birds/treatment). To euthanize the birds, we administered an overdose of barbiturate (Eskonarkon; active substance: Pentobarbitalum natricum 300 mg/1 mL) through intravenous injection at a dose of 120 mg/kg. To ensure death, we performed cervical dislocation after confirming the absence of reflex actions (i.e., pupil response). Subsequently, the birds were weighed and dissected to extract their right tibiae and humeri, which were then stored at −20°C until they underwent a three-point biomechanical test. For the biomechanical test, a Zwick and Roell Universal Testing Machine with a 2.5 kN load cell was utilized. Prior to testing, the bones were thawed for at least 24 hours at 15°C. The force was applied to the mid-shaft of the flattest side of the bones using a loading bar at a speed of 10 mm/min from which the force deformation curve was read, and the peak force in Newtons was recorded. Bone stiffness (N/mm) was calculated as the slope of the load/displacement curve, while the total work (J) done to cause fracture was determined by calculating the total area under the entire load/displacement curve. These procedures followed the ASABE Standards 2007 (ANSI/ASAE S459 MAR1992 (R2007)) as outlined by (Toscano et al., 2015).

***Statistical analysis.*** The statistical analysis was conducted using R (version 4.1.1, R Core Team, 2021) with RStudio (RStudio Team, 2021) as the graphical user interface. Linear mixed-effects models (LMM) for the ramp use behaviors and bone biomechanical properties were fitted using the 'lme4′ (Bates et al., 2015) and 'blme' (Chung et al., 2013) packages, respectively. The assumptions of homogeneity of variance and normal distribution of errors were assessed using the 'Dharma' package (Hartig and Lohse, 2021). When these assumptions were not met, appropriate data transformations were applied. Continuous explanatory variables were centered around zero, and categorical variables were contrast coded using sum contrasts, with the reference set as the mean of all groups within the variable. The p-values for each variable were calculated by model comparisons using parametric bootstrap tests performed with the 'pbkrtest' package (Halekoh and Højsgaard, 2014). Model comparisons involved reducing one single main effect or interaction at a time and comparing each reduced model to the full model. For each full model, model estimates and confidence intervals were obtained by robust covariance matrix estimation using the 'parameters' package (Bolker and Robinson, 2020), and estimated marginal means (EMM) were calculated using the 'emmeans' package (Lenth et al., 2019). We used qualitative analysis for *post-hoc* comparisons through visualizations of estimated marginal means rather than statistical tests to reduce multiple testing. Data cleaning was performed using the 'Tidyverse' package (Wickham et al., 2019) and data visualization was carried out using the 'ggplot2′ package (Wickham and Chang, 2016).

The differences in ramp use behavior between the Direct and Offset aviaries were analysed descriptively. To analyse the differences in ramp use behavior between the treatments, we used LMMs by using the number of transitions and number of active behaviors as response variables. For the mixed model analysis, we combined data from both aviaries. Both models included treatment (i.e., LED and CON) and cue status (i.e., cue-on and cue-off) as categorical variables with two levels, DoA as a continuous variable (i.e., 8, 11, 16, 23, 30, 37, 44, and 56 DoA), all two-way interactions (treatment: cue status, treatment: DoA and cue status: DoA), and a three-way interaction (treatment: cue status: DoA). An additional quadratic term for DoA was included as an explanatory variable for the model on the number of transitions. A power transformation of 2/3 was applied to the number of transitions and the number of active behaviors were square root transformed before analysis. Observation bout nested in pen crossed with date was included as a random factor in both models. Both models were fit with a Gaussian distribution.

Bone biomechanical properties (peak force required to fracture the bones, bone stiffness, and work required to fracture) were analysed separately for each bone type (tibia and humerus) using LMM with treatment as an explanatory variable, body weight as a control variable, and bird nested in pen as a random variable.

The data and code for all analysis can be found at doi.org/10.17605/OSF.IO/NM7YB

## Results

### Ramp use behavior

Most of the transitions were performed using ramps in both aviaries, with 99.51 % and 96.16 % of all transitions employing ramps in the Offset and Direct aviaries, respectively. Only 0.49 % and 3.84 % of transitions were performed by jumping or flying behavior in the Offset and Direct aviaries, respectively. Transitioning by jumping or flying showed an increase with age for the Direct aviary (DoA 8: 0.00 %, DoA 30: 5.23 %, DoA 56: 8.78 %), while jump/fly behavior for transitions did not vary depending on age in the Offset aviary (DoA 8: 0.39 %, DoA 30: 0.23 %, DoA 56: 0.93 %). In the Direct aviary, the number of transitions via jumping or flying did not significantly differ between treatments. However, in the Offset aviary, the LED groups exhibited a 16.22 % increase in jumping or flying transitions compared to the control group when summed over the entire observation period (i.e., for a total of 240 minutes observation period spread for eight days, Offset: CON = 18, LED = 16, Direct: CON = 148, LED = 172). The total number of transitions using ramps (Offset = 6916, Direct = 8024), as well as active behaviors (Offset = 3585, Direct = 6819) counted over the whole observation period (240 minutes over eight days), varied between the aviaries with more transitions and active behaviors observed in the Direct aviary.

**Fig. 2:**
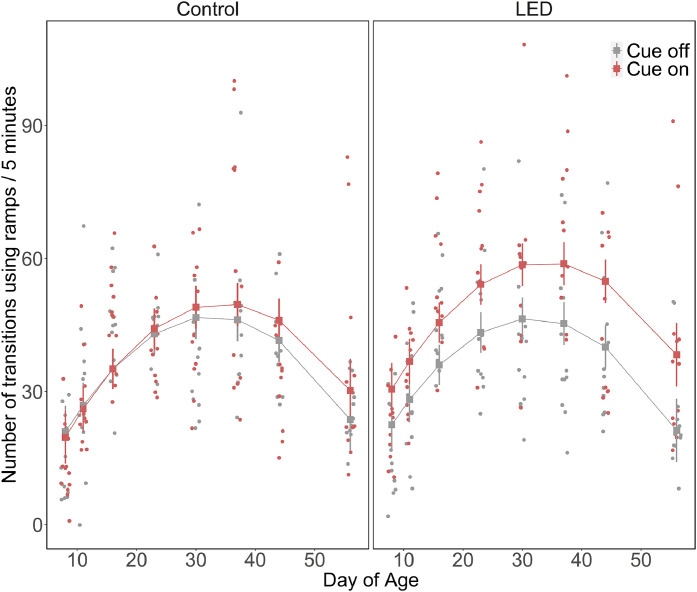
Number of transitions using ramps for the Control and LED treatments during cue on and cue off periods for all observation days. The rectangles with whiskers represent the estimated marginal means of the global linear mixed model with standard errors. The points denote the raw data.

The model parameters obtained from the mixed model analysis of the number of transitions using ramps are summarized in Table 2. The analysis revealed an effect of the interaction between treatment and cue status (Est ± CI: 1.67 [1.25, 2.22], *p*< 0.001). Birds from the LED group performed a greater number of transitions when the cues were on (EMM ± CI, 45.7 [37.4, 54.4]) compared to when the cues were off (33.9 [26.5, 41.9]) as well as cue-on (EMM ± CI, 35.7 [28.2, 43.8]) and cue-off (EMM ± CI, 34.1 [26.7, 42.1]) periods in the CON group. We also found an effect for the interaction between DoA and cue status (Est ± CI, 1.11 [0.90, 1.37], *p*= 0.003). The number of transitions increased with age reaching a peak at 3-4 WoA after which it decreased for both cue-on and cue-off periods. Overall, the number of transitions was higher when the cues were on compared to cue-off periods for all observation days. The interaction between DoA and treatment (Est ± CI, 0.98 [0.93, 1.03], *p*= 0.003) revealed that the number of transitions increased with age and peaked at 21-26 DoA after which it decreased in both treatment groups, but birds from the LED group performed more transitions than the CON group for all observation days. The three-way interaction of treatment, DoA, and cue-status did not influence the number of transitions (Est ± CI, 1.03 [0.78, 1.36], *p*= 0.83). However, we visualized the three-way interaction rather than the two-way interactions as it provided a better depiction of the patterns in the data (Fig. 2).

**Table 2:** Standardized model parameters for the linear mixed-effects models for transitions using ramps and active behaviors performed on ramps.

Transitions using ramps			
Explanatory variable	Estimate	95 % Confidence interval	p-value
Treatment	0.99	0.66, 1.45	0.27
Cue status	1.08	0.88, 1.35	0.003
DoA	1.45	1.07, 1.96	0.003
DOA^2	0.56	0.43, 0.73	0.006
Treatment:Cue status	1.67	1.25, 2.22	<0.001
Treatment:DoA	0.98	0.93, 1.03	0.003
Cue status:DoA	1.11	0.90, 1.37	0.002
Treatment:Cue status:DoA	1.04	0.78, 1.39	0.83
**Active behaviors on ramps**			
Treatment	0.96	0.38, 2.39	0.75
Cue status	1.06	0.91, 1.25	0.002
DoA	0.55	0.46, 0.65	0.002
Treatment:Cue status	1.81	1.45, 2.24	<0.001
Treatment:DoA	1.13	0.97, 1.33	0.003
Cue status:DoA	1.00	0.86, 1.17	0.77
Treatment:Cue status: DoA	0.94	0.75, 1.18	0.77

**Fig. 3:**
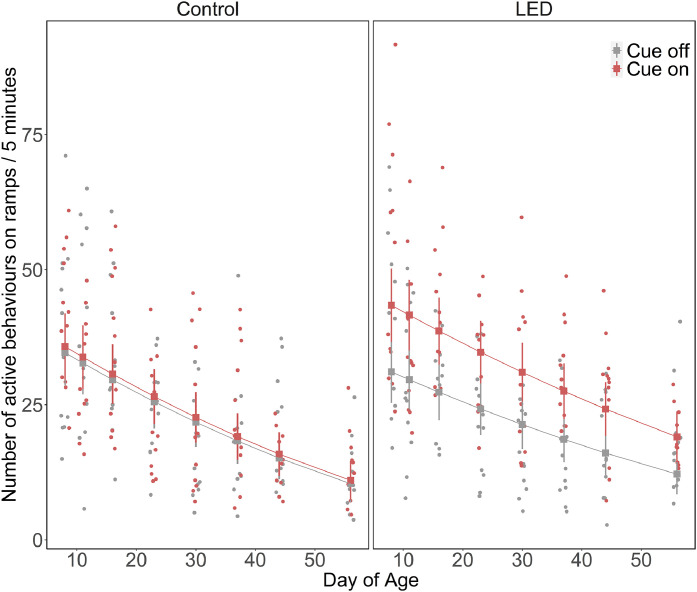
Number of active behaviors on ramps for the control and LED treatments during cue on and cue off periods for all the observation days. The rectangles with whiskers denote the estimated marginal means of the global linear mixed model with their standard errors. The points represent the raw data.

The model parameters for the number of active behaviors are provided in Table 2. The analysis revealed that the number of active behaviors was influenced by the interaction between treatment and cue status (Est ± CI, 1.81 [1.45, 2.24], *p*< 0.001). Birds from the LED group performed more active behaviors when the cues were on (EMM ± CI, 32.0 [20.1, 46.6]) compared to when the cues were off (EMM ± CI, 22.1 [12.5, 34.4]) as well as birds from the CON group (EMM ± CI: cue-on, 23.7 [13.7, 36.4], cue-off, 22.8 [13.0, 35.3]). The number of active behaviors was also influenced by the interaction between treatment and DoA (Est ± CI, 1.13 [0.97, 1.13], *p*= 0.003). The number of active behaviors decreased with increasing age for both treatments, but most number of active behaviours were observed during cue-on periods of the LED group for all observation days. The three-way interaction of treatment, cue status and DoA were visualized for the active behaviors as it provided a more accurate depiction of the data pattern (Fig. 3), although the term was not statistically significant.

### Bone biomechanics

No effect of treatment was found on peak force, total work needed to fracture the bone, and bone stiffness for either the tibiae or humeri. No effect of treatment were found for peak force (EMM ± CI (in Newton), Humeri: LED = 127.0 [121.0, 132.0], CON = 128.0 [123.0,134.0]; Tibiae: LED = 133.0 [127.0, 139.0], CON = 135.0 [129.0,141.0]), total work needed to fracture the bone force (EMM ± CI (in Joules), Humeri: LED = 0.49 [0.46, 0.52], CON = 0.48 [0.45, 0.51]; Tibiae: LED = 0.42 [0.39, 0.45], CON = 0.44 [0.41, 0.47) and bone stiffness (EMM ± CI (in N/mm), Humeri: LED = 110.0 [93.8, 126], CON = 113.0 [97.3, 129], CON = 165.0 [157.7, 174]; Tibiae: LED = 94.5 [90.3, 98.8], CON = 96.7 [92.4, 101.0]) for the different treatments. The model parameters of the LMM analysis are given in Supplement 2 (Table 1).

## Discussion

The aim of our study was to investigate if the use of a moving light cue could increase the use of ramps in the early life of laying hens in a semi-commercial rearing barn. The light cue was previously developed in small-scale experimental studies and the current experiment served as validation of the results in a commercial setting. In support of our hypothesis, the light cue increased the number of transitions using ramps as well as active behaviors performed on the ramps when the cues were on compared to when cues were off in the LED group as well as observation time slots corresponding to the cue-on and cue-off periods in CON group. However, no benefits on the bone biomechanical properties related to the increase in early life ramp use were observed. Our experiment also showed that the LED strips were commercially valid as they were cheap, easy to implement and clean, and robust.

### Biological mechanisms behind increased ramp use

The clear increase in magnitude of ramp use during the cue-on period compared to the cue-off period in the LED group as from the control group shows that the cues were effective in increasing early life ramp use. The effectiveness of the light cues in increasing ramp use may be attributed to several factors. The birds might have responded to the cues based on internal motivations associated with visual-based foraging and predatory behaviours common to modern day commercial hybrids and their ancestors (Fernández-Juricic, 2004; McBride et al., 1969). We observed the chicks following the sequentially flashing lights with their head oriented towards the ‘small moving object’ and pecking at the LED bulbs which might be indicative of following prey (Kruijt, 1964). The novelty of the cues might have also played a role in increase in ramp use. It has been shown that domestic hens use space containing novel objects more than an empty space (Newberry, 1999). The presence of LED cues could have made the ramps more attractive to chicks. The innate preference of domestic chicks for the colour red (Ham and Osorio, 2007) and objects displaying signs of animacy (Rosa-Salva et al., 2016), both intended characteristics of the light cue, likely played a role as well. The overall group response of the birds to the cues might be influenced by social facilitation as well. Meyer et al. (2021) observed that a small percentage of broiler chicks actively following the laser dot led to a widespread movement within the pen. In our own experiments, we made a similar observation where conspecifics actively following the cues on the ramp led to the recruitment of other chicks who didn't have a direct line of sight to the cues. The observations of chicks following conspecifics suggests that a portion of the increase in ramp usage can be attributed to the social facilitation tendencies of hens as well. However, as we don’t have individual level observations and know that individuals differ in their ramp use (Johny et al., 2024), there remains the possibility that the increased ramp use could be driven by a limited number of individuals.

### Benefits of cues

*Movement in aviary*: The presence of light cues led to an increased number of inter-tier transitions, implying that a greater number of birds used the upper tiers of the aviary more frequently from an early age. Early use of different aviary structures can offer more learning opportunities, leading to improved utilization of these structures at later ages. For example, birds reared with perches (Appleby et al., 1983; Faure and Jones, 1982) and ramps (Norman et al., 2021) have been shown to use these structures more in later life. Likewise, the increased experience gained in moving between different tiers with the ramps due to the cues could aid the birds in navigating the aviary more proficiently as they mature. Improving early-life ramp use by using light cues may also result in earlier access to resources distributed vertically within the aviary, which can have important welfare benefits. For example, when given access, chicks start dustbathing from one WoA (Larsen et al., 2000), and lack of proper substrate or access to the latter could induce frustration (Wichman and Keeling, 2009) and increase feather pecking (Larsen et al., 2000). Additionally, birds that had access to ramps during both rear and lay has been shown to have better plumage condition and less pododermatitis compared to those that never had access to ramps (Toscano et al., 2024).

Considering bird movement within the aviary, it's important to acknowledge strain differences. The movement patterns of white and brown birds differ, with brown birds tending to perform fewer ground and aerial movements (Pufall et al., 2021; Rentsch et al., 2023), and utilize elevated spaces less frequently (Ali et al., 2016) than white birds. Therefore, it is imperative to assess the suitability and effectiveness of these cues for brown strains as well, ensuring their applicability across different bird types.

*Enrichment:*Campbell et al. (2019) recommended research to focus on the use of enrichments for visual stimulation in poultry which should be relatively simple to use and implement within commercial facilities. It can be argued that the cues provided sensory stimulation to the hens, as we observed that the hens follow moving light cues with their head oriented towards the cue and pecking at the lit LED bulbs. The response of the birds could be due to their exploratory nature seeking novelty.

Novel objects are routinely used as environmental enrichments and have been shown to stimulate positive emotions and behaviors in poultry (Jacobs et al., 2023). Early life enrichments that the chicks readily peck, such as strings, have also been shown to reduce the severity of feather pecking in later life (Jones et al., 2000; McAdie et al., 2005). The impacts of light cues in reducing deleterious behaviors such as feather pecking is hence a possibility that should be investigated. The light cue used in our study therefore offers to be a commercially viable enrichment device that can provide sensory stimulation, increase the use of resources within the aviaries, and promote positive experiences in the early life of laying hens.

*Skeletal strength:* While an increase in ramp use can be seen as a form of load-bearing exercise that potentially enhances bone strength, our study did not reveal any differences in bone biomechanical properties between the control and light cue groups despite increased ramp use in the latter. These findings align with the results of Meyer et al. (2023), who found that an increase in walking behavior stimulated by laser pointers had no effect on breaking strength of tibia in broilers. Similarly, (Pufall et al., 2021) found that differences in wing-loading exercises, such as aerial transitions and wing-flaps, between birds reared in two aviary systems that differed in complexity had no impact on humeri breaking strength. In contrast, previous studies where differences in bone properties were identified typically involved treatment groups with more pronounced distinctions. For instance, Casey-Trott et al. (2017a) found that aviary-reared birds had greater bone breaking strength, mineral content and cross-sectional area tibial and humeral properties compared to those reared in cages. The differences in bone properties are likely attributed to the fact that aviaries offer greater opportunities for a variety of load-bearing exercises that can influence both tibia and humeri, compared to cages, which have very limited space for performing load-bearing exercises such as running and flying. In our study, the sole distinguishing factor between treatment groups was the increased use of ramps, which may not have been substantial enough to induce changes in skeletal properties. Furthermore, the birds in both our treatment groups got access to the whole aviary from DoA 8, which provided ample opportunities for dynamic load-bearing exercises, such as aerial and vertical movements and group running (personal observations), early on which has been shown to improve tibial properties (Pufall et al., 2021). Given that all the birds irrespective of treatment groups had abundant opportunities to engage in load-bearing exercises, it is possible that they reached a point where their response to further bone-loading behaviors plateaued, no longer yielding additional benefits due to increase in ramp use (Frost, 1987).

## Conclusion

In summary, our study showed that an artificial light cue that leverages the predispositions of the birds offered to be a promising tool to encourage early life ramp use in laying hens. The LED strip used in our previous and current experiments proved to be relevant in a commercial aviary setting, exhibiting several advantageous features. Despite differences in the frequency of inter-tier transitions, our research did not reveal benefits to bone properties. Further investigations into the long-term effects of the light cue, particularly regarding skeletal properties, movement within the laying house (especially following the transition from the rearing house), and its use as an enrichment device are warranted.

## Disclosures

The authors declare the following financial interests/personal relationships which may be considered as potential competing interests:

Alex Johny reports financial support was provided by 10.13039/100015221European Union. Michael Jeffery Toscano reports financial support was provided by 10.13039/501100014126European Union. If there are other authors, they declare that they have no known competing financial interests or personal relationships that could have appeared to influence the work reported in this paper.

## Appendix Supplementary materials

Image, application 1

Image, application 2

Image, video 3
