# Supplementary material for: Let there be light: Artificial light cues improve early life ramp use of laying hen chicks in a commercial aviary
Source: Poult Sci. 2025 Jul 7;104(10):105546. doi: 10.1016/j.psj.2025.105546 (PMC12329113; doi:10.1016/j.psj.2025.105546)
Supplement: Supplementary file 1 [file mmc1.docx]

Rearing management

| **Age in week** | **Lighting** | | **Temperature in degrees** | **% Relative humidity** | **Vaccination** | **Feeding chain circulations** | **Comments** |
| --- | --- | --- | --- | --- | --- | --- | --- |
|  | **Starting time** | **Hours per day** |  |  |  |  |  |
| Day 1-2 | 0 | 24 | 34 | 50 | IB primer, Marek | manual |  |
| Day 3-4 | 1 | 16 | 33 | 50 |  | manual |  |
| Day 5-7 | 1 | 16 | 32 | 50 | Paracox | manual | *Day 7* |
| 2 | 2 | 14 | 28 | 50 | IB 4/91 | 4 | *Day 14* |
| 3 | 3 | 12 | 26 | 50 | Gumboro | 5 | *Day 21* |
| 4 | 4 | 10 | 24 | 55 | Gumboro | 6 | *28.day* |
| 5 | 5 | 9 | 22 | 55 |  | 6 |  |
| 6 | 6 | 9 | 20 | 55 |  | 6 |  |
| 7 | 7 | 9 | 18 | 60 | Poulvac E-coli* | 6 |  |
| 8 | 7 | 9 | 18 | 60 |  | 6 |  |
| 9 | 7 | 9 | 18 | 60 | IB Ma5 | 6 | *Spray vaccination in the evening after lights out (turn off ventilation).* |
| 10 | 8 | 9 | 18 | 60 |  | 6 |  |
| 11 | 8 | 9 | 18 | 60 | IB 4/91 | 7 |  |
| 12 | 8 | 9 | 18 | 60 |  | 7 |  |
| 13 | 8 | 9 | 18 | 60 | AE | 7 |  |
| 14 | 8 | 9 | 18 | 60 |  | 7 |  |
| 15 | 8 | 9 | 18 | 60 | Pulvac E-Coli | 7 | *If animals well recovered already 10 day after AE otherwise 14 day after AE 🡪Drinking water vaccination* |
| 16 | 8 | 9 | 18 | 60 |  | 8 |  |
| 17 | 7 | 10 | 18 | 60 | IB vaccine | 8 |  |
| 18 | 7 | 10 | 18 | 60 |  | 8 |  |
