# Supplementary material for: Let there be light: Artificial light cues improve early life ramp use of laying hen chicks in a commercial aviary
Source: Poult Sci. 2025 Jul 7;104(10):105546. doi: 10.1016/j.psj.2025.105546 (PMC12329113; doi:10.1016/j.psj.2025.105546)
Supplement: Supplementary file 2 [file mmc2.docx]

**Supplement 2**


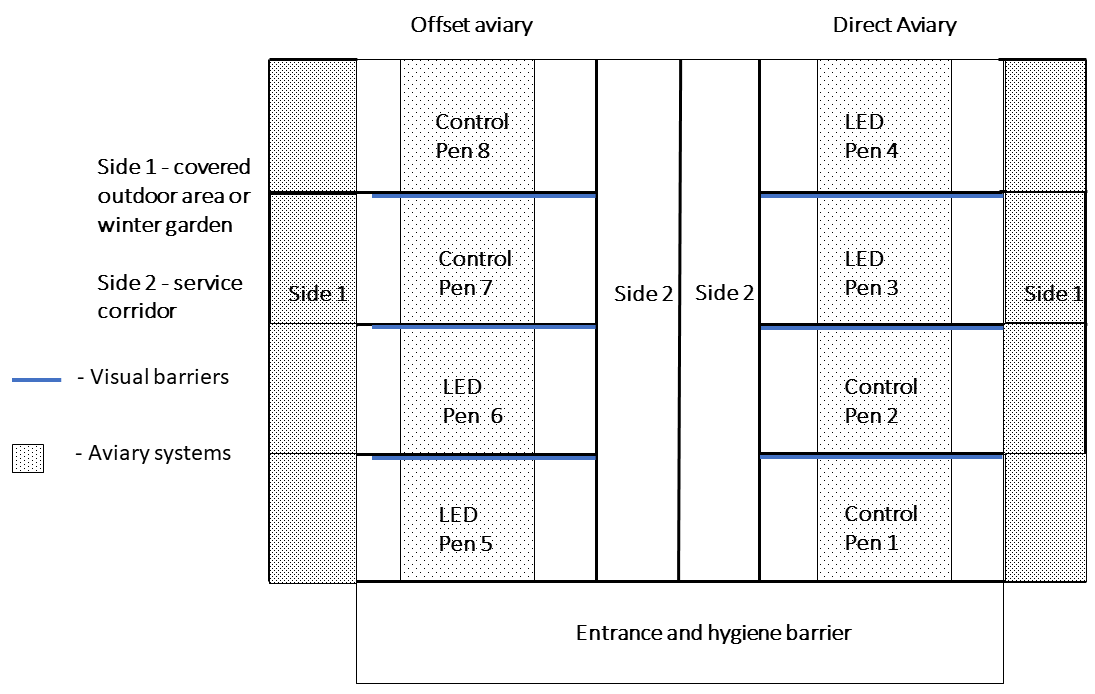
**Figure 1: Treatment pen allocation for the aviaries**

**Table 1: Standardized model parameters from linear mixed model analysis for bone biomechanical properties.**

| Bone biomechanical property | Bone type | Estimate for treatment | 95% confidence interval | p-value |
| --- | --- | --- | --- | --- |
| Stiffness | Tibia | 0.79 | 0.58, 1.07 | 0.83 |
| Peak force |  | 0.85 | 0.62, 1.17 | 0.74 |
| Work to fracture |  | 0.76 | 0.63, 0.91 | 0.11 |
| Stiffness | Humerus | 0.86 | 0.50, 1.45 | 1.00 |
| Peak force |  | 0.88 | 0.73, 1.07 | 0.72 |
| Work to fracture |  | 0.88 | 0.64, 1.21 | 0.94 |
